# Locomotion-Related Oscillatory Body Movements at 6–12 Hz Modulate the Hippocampal Theta Rhythm

**DOI:** 10.1371/journal.pone.0027575

**Published:** 2011-11-15

**Authors:** Anders Ledberg, David Robbe

**Affiliations:** 1 Department of Information and Communication Technologies, Center for Brain and Cognition, Universitat Pompeu Fabra, Barcelona, Spain; 2 Department of Systems Neuroscience, Institut d'Investigacions Biomèdiques August Pi i Sunyer (IDIBAPS), Barcelona, Spain; Instituto de Neurociencias de Alicante UMH-CSIC, Spain

## Abstract

The hippocampal theta rhythm is required for accurate navigation and spatial memory but its relation to the dynamics of locomotion is poorly understood. We used miniature accelerometers to quantify with high temporal and spatial resolution the oscillatory movements associated with running in rats. Simultaneously, we recorded local field potentials in the CA1 area of the hippocampus. We report that when rats run their heads display prominent vertical oscillations with frequencies in the same range as the hippocampal theta rhythm (i.e., 6–12 Hz). In our behavioral set-up, rats run mainly with speeds between 50 and 100 cm/s. In this range of speeds, both the amplitude and frequency of the “theta” head oscillations were increasing functions of running speed, demonstrating that the head oscillations are part of the locomotion dynamics. We found evidence that these rhythmical locomotor dynamics interact with the neuronal activity in the hippocampus. The amplitude of the hippocampal theta rhythm depended on the relative phase shift with the head oscillations, being maximal when the two signals were in phase. Despite similarity in frequency, the head movements and LFP oscillations only displayed weak phase and frequency locking. Our results are consistent with that neurons in the CA1 region receive inputs that are phase locked to the head acceleration signal and that these inputs are integrated with the ongoing theta rhythm.

## Introduction

Locomotion in bipedal and quadrupedal animals is characterized by rhythmical coordinated movements of the limbs. Locomotor rhythms can additionally be observed throughout the body. In particular, the head moves vertically in a sinusoidal manner, completing two cycles for each step cycle [1], [2]. On the one hand, the regular repetition of the elementary movements of locomotion (e.g., step cycle) is believed to serve a navigational function by helping to estimate traveled distance, a process referred to as path integration [3]. On the other hand, the oscillations of the body that accompany locomotion can potentially interfere with sensory processing. For instance, when animals walk or run, the ‘sensors’ located on the head (e.g. the eyes) are moving up and down and it is fundamental to distinguish sensory inputs due to these self-generated movements from those due to real movements of the environment. From these functional implications of locomotor activity, it is reasonable to assume that the oscillatory dynamics of locomotion are integrated in neuronal activity in brain regions important for spatial navigation and high-level sensory processing.

The theta frequency oscillations of the hippocampal local field potential (LFP) is the main brain rhythm expressed when mammals actively move in their environments [4]. A large variety of functions have been attributed to theta but most views agree that it participates in a high-level processing of sensory information [5], reviewed in [6]. Moreover, several studies support the hypothesis that theta, by imposing temporal structure on neuronal activity, plays a crucial role in navigation and spatial memory [7]–[15]. Despite the prominence of theta when animals walk or run, and the relevance of the rhythmical dynamics of locomotion for spatial navigation and sensory integration, the relation between hippocampal theta and the movements of locomotion is elusive.

Here we investigated if and how the hippocampal theta rhythm is influenced by the periodic movements of locomotion. We combined precise behavioral quantification, using head-mounted miniature accelerometers, with simultaneous recordings of the CA1 hippocampal LFP in freely running rats. We found that during running, the rats' heads oscillate in the vertical plane (up and down) at 6–12 Hz in a speed-dependent manner and we used this head acceleration signal as a proxy for any rhythmical locomotion-related signal. We further found that this locomotor rhythm interacted with the amplitude of the hippocampal theta rhythm. Theta amplitude was maximal (minimal) when the phases of theta and head oscillations were similar (dissimilar). On the contrary, despite striking similarity in their oscillation frequency, hippocampal theta and vertical head movements displayed only marginal phase-locking and coherence. These results are consistent with a neuronal input to the hippocampus that is phase locked to the head movements and which is integrated with the ongoing theta rhythm.

## Materials and Methods

All animal procedures were conducted in accordance with standard ethical guidelines (European Communities Directive 86/60-EEC) and were approved by the local ethical committee (Comité d'Experimentació Animal, Universitat de Barcelona, Ref 520/08).

### Hippocampal LFP and head acceleration recordings in running rats

Silicon probes (from Neuronexus, Rat 2 and 3) or an array of 4 tetrodes (Rat 1) were implanted above the right hippocampus under deep isoflurane anesthesia. Craniotomies were centered at the stereotaxic coordinates of −3.2AP and 2.5ML relative to Bregma. Silicon probes/tetrode arrays, attached to movable microdrives and oriented along the transverse axis of the hippocampus were implanted in the deep layers of the parietal cortex (−1.4DV below brain surface). One ground and one reference screws were implanted in the bone above the cerebellum. The microdrive assembly was protected by a copper mesh that also served as a local Faraday cage and support for the accelerometer (see below). After complete recovery from the surgery (1 to 2 weeks), the electrodes were slowly lowered toward the hippocampal CA1 pyramidal layer. The pyramidal layer of the CA1 area was identified by the presence of sharp wave/ripple complexes associated with quiet immobility, and burst of unit activity [16], [17]. In the data presented here, electrodes were localized just above the pyramidal layer as attested by the presence of ripples and the absence of bursts of unit activity or downward sharp waves (not shown).

**Figure 1:**
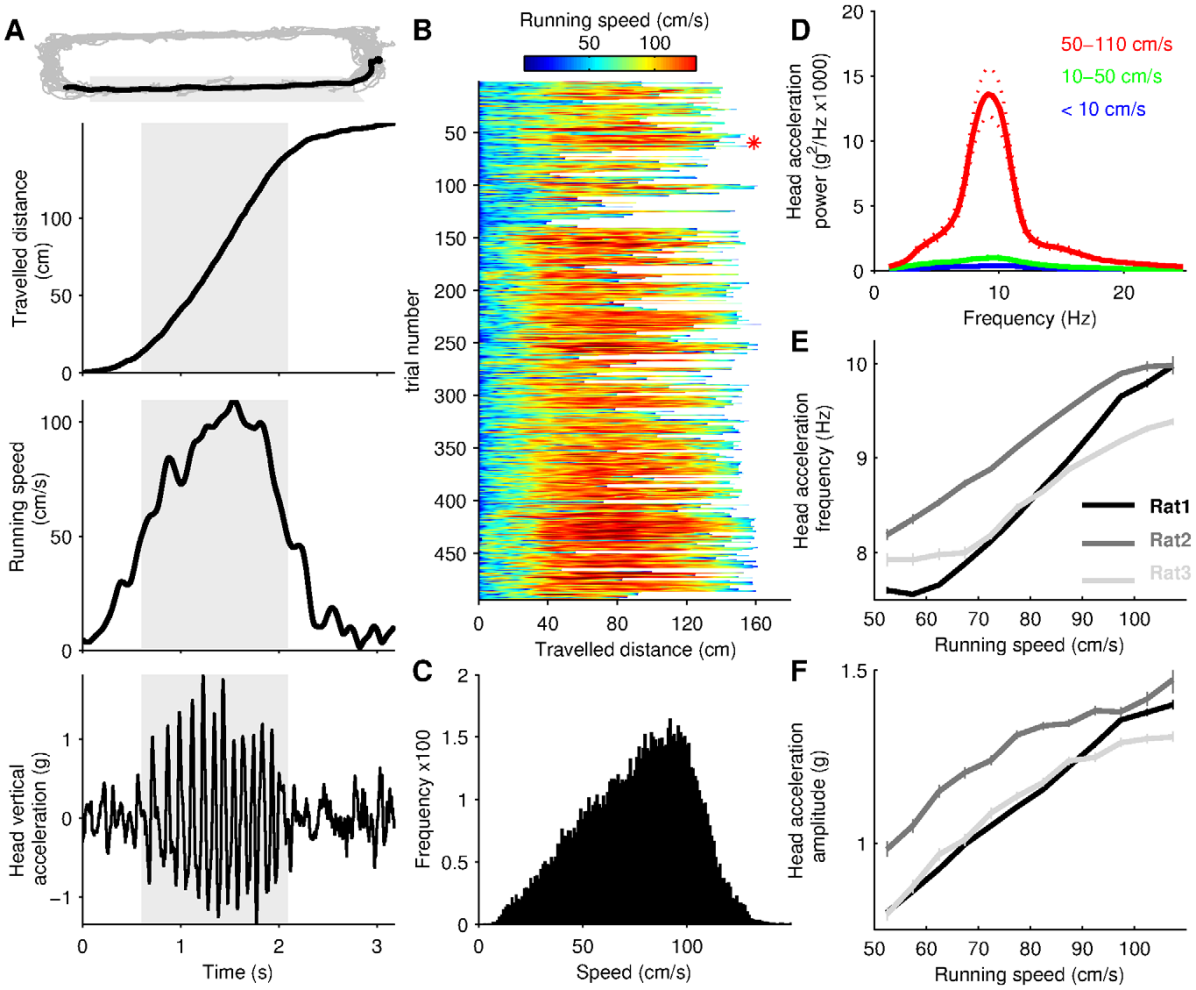
Vertical oscillations of the head at 6–12 Hz when rats run. (A) Behavioral measures from a representative trial (a continuous run across the maze, see Materials and Methods). Upper panel shows the trajectory of the animal during a complete behavioral session (light gray) and a single trial (black). The three lower panels show, from top to bottom, traveled distance, running speed, and vertical head acceleration during this trial. Shaded gray areas correspond to time at which running speed is superior to 50 cm/s. (B) Running speed versus traveled distance for all the trials analyzed in a single animal (Rat 2). Red star represents the trial shown in A. (C) Distribution of the running speeds shown in B. (D) Power spectrum of the vertical head acceleration during slow (black), intermediate (blue) and fast (red) running speeds. Dotted lines shows 95% interval confidence. Traces are averages of power spectra from all the recorded sessions for Rat 2. (E,F) Relationship between running speed and vertical acceleration frequency (E) and amplitude (F), for the 3 rats. Data in E–F are means +/− sem computed over data from all sessions.

Once ripples were detected, the rats were placed under a controlled water restriction protocol (weight between 85 and 90% of the normal weight) and trained to run back and forth in a rectangular maze for water reinforcement. The maze (160×40 cm) had 45 cm high lateral walls. An additional wall placed in the center of the arena created a 15 cm wide rectangular track along which the rat could run in any direction. Drops of water were delivered through two small tubes coming out from the centers of the two shortest walls of the maze (Fig. 1A). Each time the animal crossed the maze, a single drop of water was delivered in the water tube by opening a solenoid valve controlled remotely via a computer interface (LabView). Importantly, reinforcement was not conditional on the speed or the path of the animals. Rats learned very quickly (one to two days) to run back and forth in the maze to collect water and performed reliably, crossing the maze at fast speed (e.g. Fig. 1A). One to eight recording sessions, each lasting 10 to 20 min, were performed daily. The length and number of recording sessions varied depending on the animals motivation and quality of the recordings. The data analyzed here were recorded in 59 sessions performed across 14 days (Rat 1: 25 sessions in 7 days, Rat 2: 10 sessions in 3 days, Rat 3: 24 sessions in 5 days).

Measurements of linear head acceleration were made with a triple axis miniature accelerometer mounted on a breakout board of dimension 1.8 by 1.8 cm (SparkFun Electronics, ADLX 335 for Rat 1 and 3 and MMA7260Q for Rat 2). A small cable jumper was custom-built to power the accelerometer (+3.5 V) and convey X-Y-Z acceleration signals (300 mV/g for ADLX 335 and 200 mV/g for MMA7260Q) to the recording system. For precise positioning of the accelerometer on the recording headstage, the following procedure was followed: Two plastic screws were inserted head down through two small holes drilled in the breakout board and the breakout board/screw assembly was secured with wing nuts. At the end of the surgical electrodes implantation, the breakout board/screw assembly was positioned behind the top of the Faraday cage under stereotaxic control, so that the X, Y and Z axes of the accelerometer were parallel to respectively, the anteroposterior, mediolateral and dorsoventral axes of the rats head. The heads of the plastic screws were cemented to the outside face of the Faraday cage. The wing nuts were unscrewed and the breakout board was slid upward out of the screws. This simple design allowed easy and reliable re-positioning of the accelerometer for each recording session. In principle, it is possible to calculate the vertical head displacement from the Z-axis acceleration signal (by integrating the signal twice), in practice this double integration tends to accumulate errors and make the resulting position signal more noisy than the ‘raw’ acceleration signal. Here, we therefore used the acceleration signal for illustrations and comparisons with hippocampal neuronal activity. Furthermore, during running, the rats heads display pitches and rolls that we could not accurately compensate for. The Z-axis acceleration signal recorded is therefore an underestimate of the vertical head accelerations performed by the animal. The Z-axis acceleration signal should be considered primarily as a proxy for rhythmical locomotion-related dynamics and not as providing an exact measure of head movements.

Wide band (0.1 Hz–8 kHz) neurophysiological signals (amplified 1000 times via a Plexon VLSI headstage and a Plexon PBX2 amplifier) and acceleration signals (1–50 Hz) were digitized at 20 kHz on two synchronized National Instruments A/D cards (PCI 6254, 16 bit resolution). LFP and final acceleration signal acceleration were obtained by downsampling the acquired signals at 1250 Hz. To track the positions of the animal, one LED located above the accelerometer was recorded using a ceiling-mounted digital video camera operating at 25 frames per second.

### Trials isolation

To isolate epochs during which the animals were running, instantaneous running speed was estimated from the video tracking system and smoothed with a Gaussian kernel of standard deviation 100 milliseconds. Epochs during which the animals crossed the maze with a speed superior to 10 cm/s for at least one second were considered *trials*. One trial was generally identified each time the animal crossed the maze. We analyzed 944 trials in 25 sessions in Rat 1, 494 trials in 10 sessions in Rat 2 and 868 trials in 24 sessions in Rat 3.

### Power spectrum and coherence analysis

The spectral and coherence analyses were made using a multitaper approach [18] as implemented in the Chronux toolbox (http://chronux.org/, [19]). Both LFP and acceleration signals were digitally high-pass filtered at 1 Hz.

For Fig. 1D, the head acceleration signal was divided according to the running speed in each behavioral session. The three power spectrum density functions shown are averages calculated over all the sessions recorded in Rat 2 (n = 10). For Fig. 1E, a time frequency spectrogram was computed using non-overlapping windows of 0.5 second. This analysis was applied to the head acceleration signal during all the trials. For each window, weighted mean frequency between 6 and 12 Hz and average running speed were determined. For Fig. 1F, the amplitude of the acceleration signal ((max-min)/2) was computed using the same data windows as above.

Power spectrum and coherence analyses of LFP and acceleration signals were computed from all data coming from trials isolated as described above.

### Phase locking and phase-dependent modulation quantification

To investigate if the amplitude of the LFP was modulated according to its phase lag relative to the oscillatory head movements, we first isolated epochs during which the head displayed prominent oscillatory movements. For this, the Z-axis acceleration signal in the trials was band-pass filtered between 4 and 12 Hz. The filtered signal was squared, smoothed with an averaged filter of 160 milliseconds and standardized. Oscillatory epochs were defined as the time during which the standardized signal was superior to two standard deviations for at least half a second. Such oscillatory epochs were detected in 758 trials in Rat 1 (80% of all trials recorded), 314 in Rat 2 (64%), and 438 trials in Rat 3 (50%). Next, we detected the peaks of the head acceleration signal in the “oscillatory segments”. At the time of the acceleration peaks, the instantaneous phase of the theta oscillations was extracted using the Hilbert transform of the 4–12 Hz filtered LFP. The amplitude of the LFP was taken as the root mean square (rms) of the LFP in 250 milliseconds windows centered around the time of the acceleration peak. Each acceleration peak was therefore associated with two values: the instantaneous phase and amplitude of theta.

To investigate the relationship between theta amplitude and the phase lag between theta and head oscillations, the amplitude values were binned in phase-lag windows of 30 degrees and averaged. To test the significance of the amplitude modulation, we used a jittering procedure coupled with point-wise nonparametric statistics. Surrogate data sets were generated by randomly shifting, for each trial separately, the time of the acceleration peak relative to LFP windows by a value between −55 and +55 milliseconds (110 milliseconds is the approximate period of theta and head acceleration oscillations in our data). All the acceleration peaks of the same trial were identically shifted to maintain a similar intra-trial bias in the surrogates data. This process was repeated 500 times. Next, average theta amplitude versus theta-head phase lag was extracted for each surrogate data set. The amplitudes versus phases of the 500 surrogate data sets were sorted to obtain a pointwise acceptance level of 99%. Similar amplitude modulation results were obtained if maximum amplitude, power of the LFP in the theta band or amplitude of the LFP Hilbert transform were used (not shown).

To test the hypothesis that the phases of the LFP and head acceleration are locked, we computed the phase differences of the two signals in sliding windows. For continuous data, phase differences are only meaningful when oscillations have similar frequency (or if one signal's frequency is a harmonic of the other's). In the oscillatory epochs isolated as described above, a 250 milliseconds long window was advanced in steps of 50 milliseconds. In each window, the instantaneous phases of the LFP and head acceleration were quantified using the Hilbert transform of the 4–12 Hz filtered signals. The mean phase difference was then calculated for each window. Additionally, the frequency of the LFP and acceleration in each window was extracted by finding the peak frequency of their discrete Fourier transform. For each analysis, this data processing generated 12355 data windows in Rat 1 (from 758 trials in 25 sessions), 3795 windows in Rat 2 (317 trials/10 sessions) and 5601 windows in Rat 3 (438 trials/24 sessions). Histograms were obtained from data windows in which the oscillation frequencies of the LFP and head acceleration did not differ by more than 1 Hz (resulting in 7410, 2774 and 3263 windows in respectively Rat 1, 2 and 3). We then tested if the phase difference distributions were significantly non-uniform. Statistical tests assuming that the data are sampled from von Mises distribution (e.g., Rayleigh test) could not be used here because the phases of windows extracted from the same trial are not independent. To test the significance of the phase distributions obtained in the three rats we proceeded as follows: Surrogate data sets were generated by randomly shifting, for each trial, the time of the acceleration windows relative to LFP windows by a value between −55 and +55 milliseconds. This process was repeated 500 times. Next, we reasoned that, to be significant, the mean vector length of the real phase distribution should exceed the 95^th^ percentile of the surrogate distribution. Direction and length of mean result vectors along with other operations on circular data were calculated using the Circular Statistical Toolbox for Matlab [20].

## Results

Three rats were trained to collect drops of water alternatively delivered at the two extremities of a 1.6 meter long rectangular maze (Fig. 1A). After one or two days of training, the rats performed this simple task reliably by crossing the maze at fast speeds (Fig. 1A–C, mean speed in cm/s +/− std: 75.4+/−40.1 for Rat 1, 76.3+/−25.6 for Rat 2; 101+/−40.4 for Rat 3). We quantified the dynamics of locomotion by measuring vertical acceleration of the head (Materials and Methods). When rats ran at speeds between 50 and 100–110 cm/s, the acceleration signal displayed prominent oscillations (Fig. 1A, lower panel), indicating that the heads moved with a regular oscillatory pattern in the vertical plane (up and down). This running speed range was consistently reached over a large portion of the maze (Fig. 1A–C), in agreement with other studies using similar behavioral settings [21]–[25], and was associated with trotting (not shown), as expected from kinematic studies of rat locomotion [25]. We next performed a power spectrum analysis of the acceleration signal during slow (speed<10 cm/s), intermediate (10–50 cm/s) and fast running speeds (50–110 cm/s). This analysis showed that the regular head movements shown in Fig. 1A are primarily associated with running at fast speeds and they have a frequency ranging from 6 to 12 Hz (Fig. 1D). We additionally noticed that rats occasionally run at speed above 110 cm/s by galloping. During these runs, the head acceleration signal did not display regular oscillations at 6–12 Hz but a more irregular pattern reflecting the variability of the galloping gaits (symmetric or trailing leg gallop, not shown, [21]). Next, we investigated how the frequency and amplitude of the 6–12 Hz head oscillations were related to running speed. When rats run above 50 cm/s there was a monotonous, almost linear, relationship between these two parameters and speed (Fig. 1E,F), confirming that the oscillatory head movements are part of the dynamics of locomotion [1], [2], [26].

**Figure 2:**
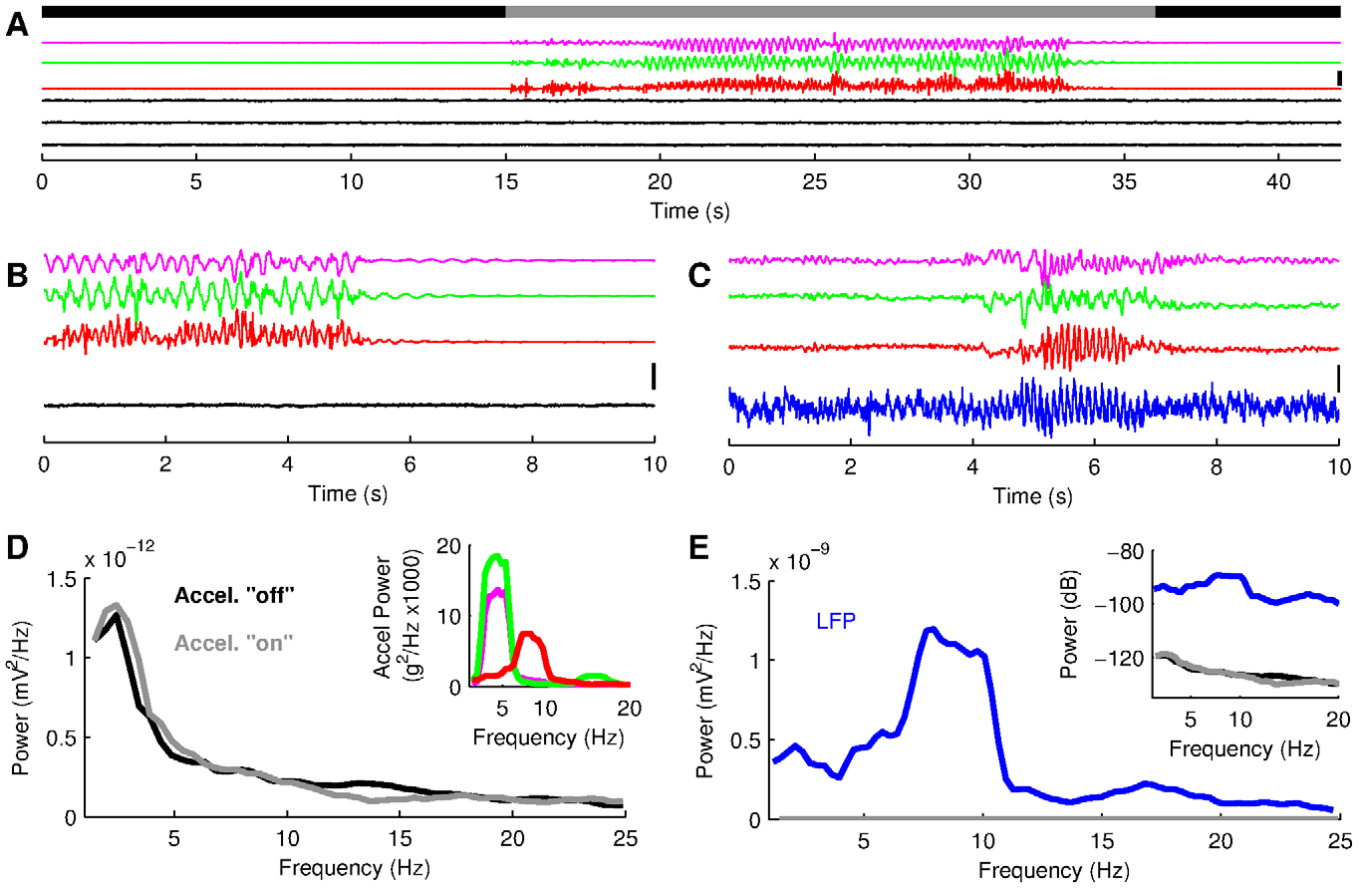
Signals generated by the accelerometer do not interfere with the neurophysiological recording apparatus. (A) Recording from a saline container (black traces) while the accelerometer is moved manually in an oscillatory manner at proximity of the recording sites. The time intervals during which the accelerometer were immobile and moved are represented respectively by the black and gray segments of the upper bold line. The colored traces show the signals generated by the accelerometer (pink, green and red for respectively the anteroposterior, mediolateral and dorsoventral axes). (B) 10 seconds epoch from A. (C) 10 seconds recording epoch taken around a single trial performed by Rat 1. Blue trace represents CA1 LFP. (D) Spectral density functions from the recording shown in A, during and around movements of the accelerometer. Inset shows the spectral density functions of the 3 accelerometer signals when the accelerometer was moved. (E) Spectral density functions of the hippocampal LFP (from data shown in C) and of the saline recordings (same than D). Note the different y-axis scales in D and E. Inset shows the same data using a logarithmic scale for the y-axis, to visualize saline recording power spectrum. The vertical scale bars in A, B and C represent 0.5 mV for the LFP and saline recordings and 1.6 g for the acceleration signals respectively.

Earlier studies have shown that hippocampal theta is related to sensorimotor activities [4], [5], [27]. We set out to investigate this relationship in more detail by comparing the head acceleration signal with the simultaneously recorded hippocampal LFP. We first verified that the electrical signals generated by the accelerometer did not interfere with our neurophysiological recording set-up. For this purpose, we recorded from a saline recipient while moving the accelerometer in an oscillatory manner (Fig. 2A). In this set-up, the distances between accelerometer, headstage preamplifier and electrode were similar to that in the neurophysiological recordings. Visual inspection and power spectrum analysis of the saline recordings showed that there is no noticeable influence of the accelerometer on our recording system (Fig. 2A,B,D,E).

**Figure 3:**
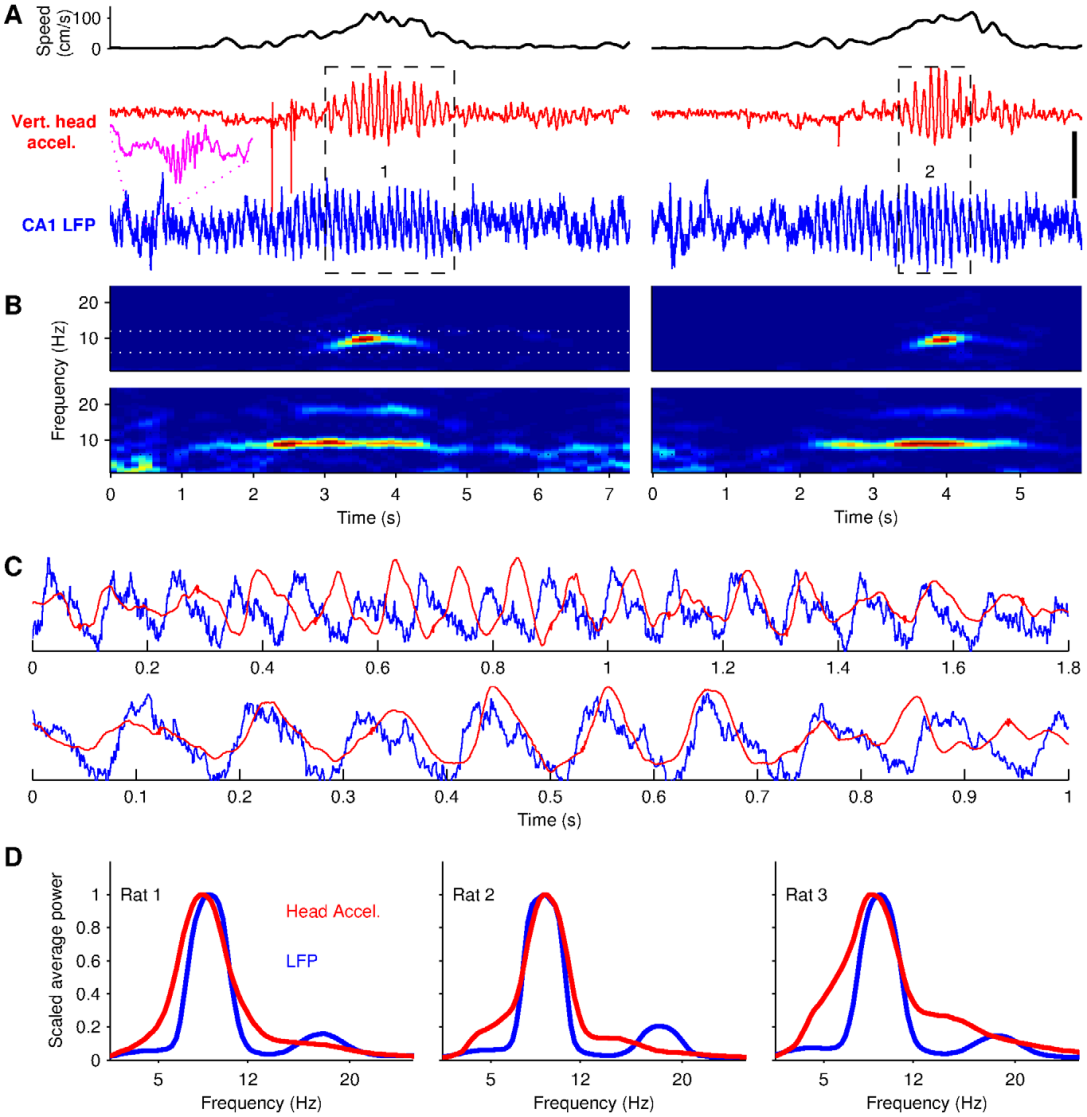
Head acceleration and LFP oscillate at theta frequency during running. (A) Simultaneously recorded vertical acceleration of the rat's head (red) and CA1 hippocampal LFP (blue), before, during and after two runs across the maze (Rat 2, same recording session). Top black traces show the running speed of the animal. Magenta inset shows a ripple. Data in the dashed rectangle are magnified in C. (B) Time-frequency power spectra of the acceleration (upper row) and LFP (lower row) signals shown in A. White dotted lines show the limits of the 6–12 Hz theta band. (C) Superposition of LFP and acceleration signals (scaled) when head oscillations are prominent. Upper and lower traces are taken respectively from dashed rectangles 1 and 2 in A. (D) Average power spectra of the LFP (blue) and head acceleration (red) during all the trials recorded for the three rats (Rat 1 to 3 are shown from left to right). The vertical scale bar in A represents 0.5 mV and 2.4 g for, respectively, the LFP and acceleration signal.

To investigate the relation between hippocampal theta oscillations and oscillatory head movements, LFP recordings were performed just above the pyramidal layer in the CA1 area as attested by the presence of ripples (inset Fig. 3A) and lack of bursts of unit activity characteristic of the pyramidal layer and downward sharp waves characteristic of the radiatum layer (not shown). Examination of representative segments of the raw LFP revealed that theta typically emerged before and terminated after the head oscillations (Fig. 3A,B). Analysis in the frequency domain (Fig. 3B) and detailed visual inspection of illustrative trials (Fig. 3C) indicated that the two signals have strikingly similar frequencies. A spectral analysis of the data from all trials in the three animals confirmed these observations, showing an almost perfect overlap of the main frequency bands in the two signals (Fig. 3D). Additionally, the phases and amplitudes of theta and especially of the head acceleration signal show substantial uncoordinated variability on a short timescale (Fig. 3C). We next characterized the interactions between the two signals in more detail.

The hippocampal theta rhythm can be observed without simultaneous rhythmical body movements (e.g. Fig. 3A,B, see [6] for review) and we therefore designed our analysis assuming that the head acceleration acts as a perturbation of the theta rhythm. (There are many neural structures where the activity is phase locked to the head acceleration (e.g. in the vestibular system and the motor system). We use the ‘raw’ head acceleration signal as proxy for any of these locomotion-related neuronal signals and mention some possible pathways by which these signals can alter hippocampal activity in the Discussion). To a first approximation, the hippocampal theta rhythm can be modeled as a sinusoid with slowly changing frequency and amplitude (see e.g. Fig. 3C). This means that it is well characterized by three parameters: amplitude, frequency, phase. The head acceleration could in principle perturb any of these parameters. We will first consider how the theta amplitude is influenced by the head oscillations and subsequently look for modulations of frequency and phase.

**Figure 4:**
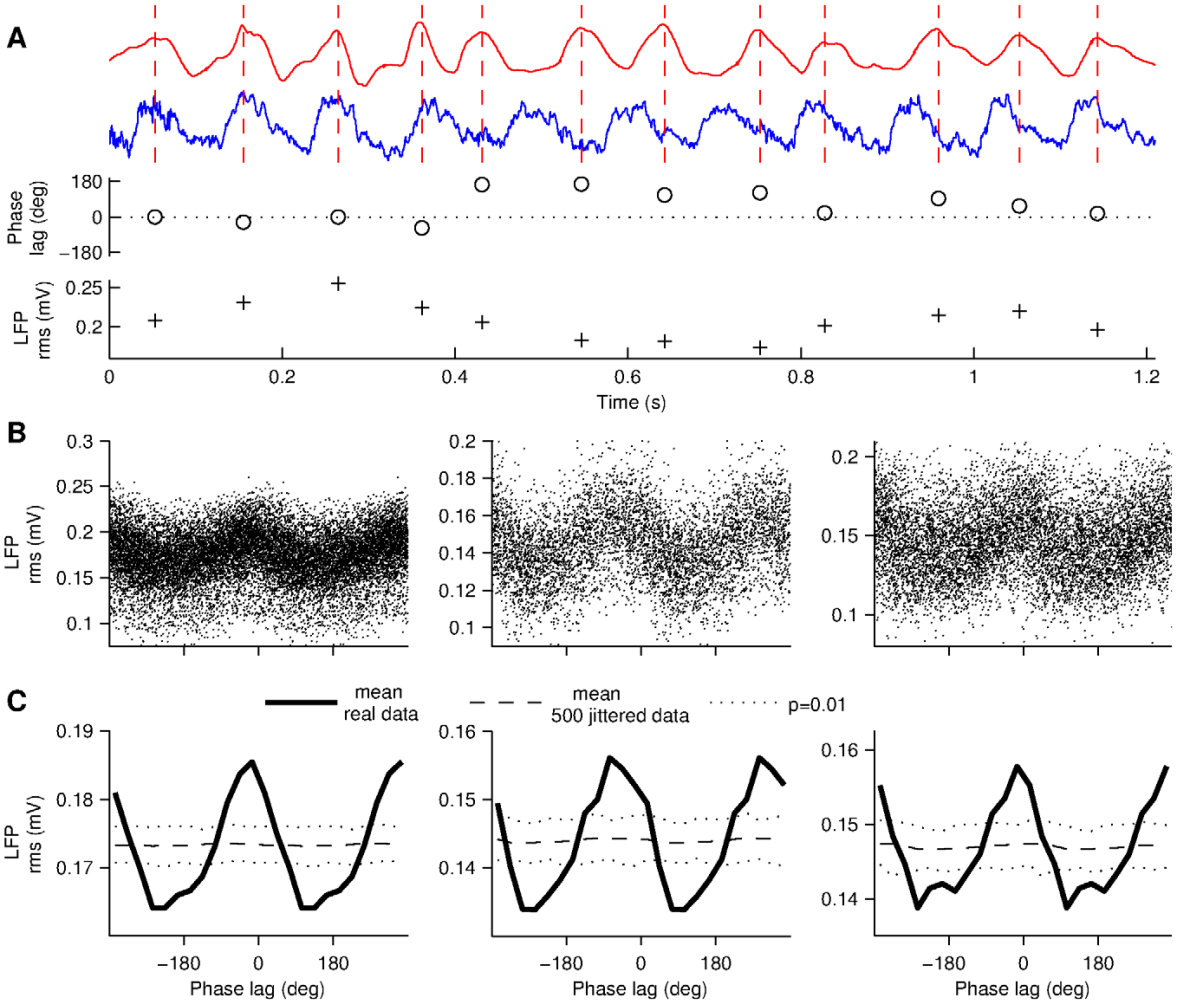
Phase-dependent modulation of theta amplitude by head movements. (A) Upper traces illustrate the detected peaks of the acceleration signal during prominent oscillatory head movements. Circles (o) show the instantaneous phase lag between the two signals, at the time of the peak. Plus signs (+) show the amplitude (rms) of the LFP in 250 milliseconds long windows centered around the peaks. Note that a trial with very irregular head acceleration signal was purposely chosen to illustrate the analysis method. (B) Theta amplitude versus head-LFP phase lag for all the peaks detected in Rat 1, 2 and 3 (left to right). (C) Average amplitude modulation of values in B for the three rats. Dashed line show average of jittered surrogate data. Surrogate data were used to construct a pointwise acceptance level of 99% (dotted lines).

To investigate locomotion-related modulations of theta amplitude, we tested if the amplitude of theta depended on the relative phase difference with the head acceleration signal. First, head acceleration peaks were isolated in data segments having prominent oscillatory movements (Materials and Methods, Fig. 4A). At the time of the acceleration peaks, the instantaneous phase and amplitude of the theta oscillations were determined. The amplitude of theta was taken as the root mean square (rms) value of the LFP in 250 milliseconds windows centered around the acceleration peak. Each acceleration peak was therefore associated with 2 values: the instantaneous phase of theta and its “local” amplitude. The phase lag between theta and head acceleration had a strong effect on the rms (Fig. 4B,C). The observed effect, higher theta amplitude for small phase lags, is clearly visible in the population data in all three rats (Fig. 4B) and the effect is highly statistically significant (Fig. 4C). These amplitude-modulation results are consistent with that hippocampal cells receive inputs phase-locked to the head acceleration, and since these inputs have similar frequency as the hippocampal theta rhythm, a phase dependent modulation occurs (see Discussion).

**Figure 5:**
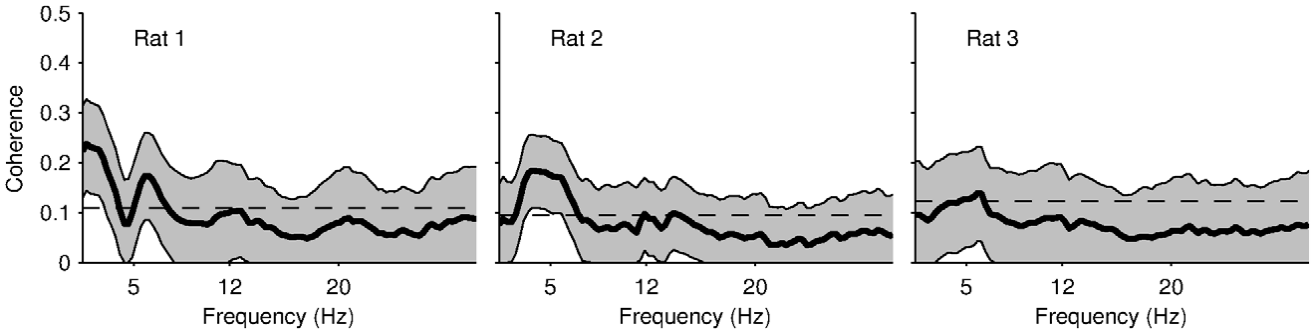
Head and LFP oscillations display insignificant coherence in the theta frequency band. Average coherence between acceleration signal and LFP. Dashed lines and shaded areas show 95% confidence interval under the hypothesis of independence.

**Figure 6:**
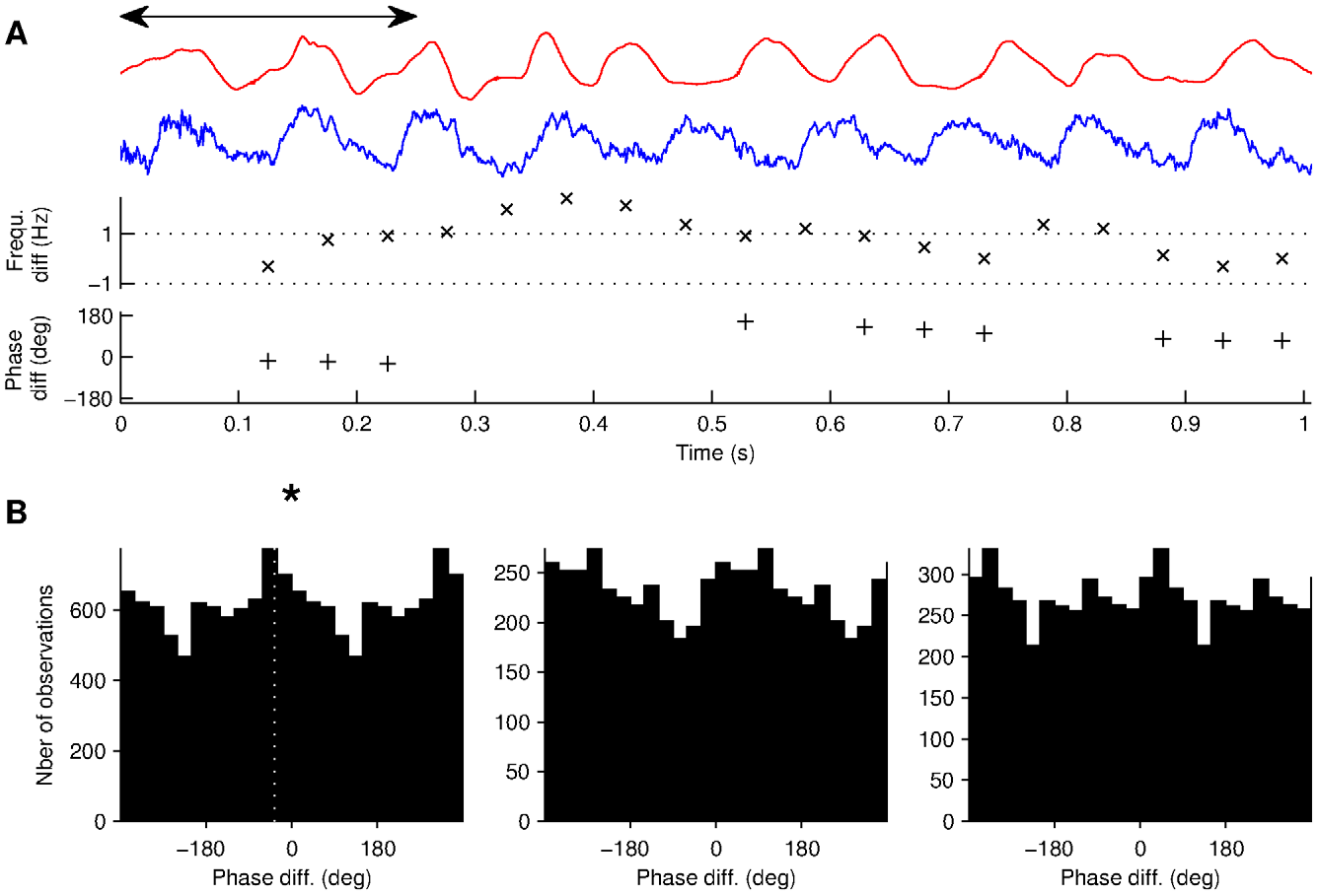
The phases of the head and LFP oscillations display marginal phase locking across animals. (A) Illustration of the method. A 250 milliseconds long window (double arrowhead) was advanced in step of 50 milliseconds during epochs of prominent head oscillations. Red and blue traces show respectively the acceleration signal and LFP (same illustrative epoch as in Fig. 4A). Crosses (x) show the frequency differences for each window. Plus signs (+) show the phase difference for windows of similar frequency. (B) Phase difference distribution for Rats 1 to 3 (left to right). The star indicates statistical significance versus jittered surrogate data (see Materials and Methods). Dotted line shows the mean direction of the phase difference distribution for Rat 1.

Another possible type of interaction between putative locomotion related signals and hippocampal theta rhythm is synchronization. Since the frequency of the head oscillation was very similar to that of the theta (Fig. 3), the head movements might indeed entrain the theta rhythm. If this was the case, we should see a relationship between the frequencies, amplitudes and phases of the two signals [28]. The instantaneous frequencies of head oscillations and hippocampal theta were weakly positively correlated in all three rats (p<0.05, permutation test) but this correlation could be partly explained by running speed [29]. Indeed, after partialling out running speed, the correlation was significant in only two of the three rats (with partial correlation coefficients: 0.08, and −0.06). The small magnitude of these correlation coefficients, and their different signs, indicate that the frequencies of the two oscillations were largely independent. A similar result was obtained for the correlation between the amplitudes of the LFP and acceleration signal (i.e., weak correlation largely explained by running speed). We next used coherence analysis to investigate if there was a linear relationship between the phases and amplitudes of the two oscillations. Although there seemed to be a weak coherence at frequencies below 5 Hz, there was no statistically significant coherence in the theta band (Fig. 5). Coherence analysis is essentially a correlation analysis of the Fourier coefficients at each frequency. Given that the frequency of the head oscillation changes substantially during one ‘trial’ and that the amplitudes of the two signals are modulated (e.g. Fig. 3C), coherence analysis might not be the most sensitive tool to discover a phase locking between the two signals. Consequently, to directly test if the two signals were phase locked, the phase differences between theta and head acceleration oscillations were determined using sliding windows (Materials and Methods, Fig. 6A). For each rat, the distribution of theta-head phase differences are shown in Fig. 6B. The presence of small peaks and troughs in the three histograms suggested that the phase difference values are not uniformly distributed but rather show weak unimodal distribution, i.e. phase locking. The mean direction of the relative phase distributions was −36°, 79° and 6° for respectively Rat 1, 2 and 3. The length of the mean resultant vectors was 0.066, 0.068 and 0.031 for, respectively, Rat 1, 2 and 3. The statistical significance of these deviations from a uniform distribution were assessed by randomly jittering the time of the acceleration windows relative to the time of the LFP windows and comparing for each rat the length of the mean resultant vector of the real data with those of the jittered data (Materials and Methods). The result of this procedure showed that the small non-uniformity in the distributions of the phase differences were significant in Rat1 (upper 95^th^ percentile of the surrogates were 0.057, 0.1 and 0.07 for respectively, Rat 1, 2 and 3). Altogether these results indicate that the phase and frequency of hippocampal theta are relatively unaffected by the oscillatory dynamics of locomotion. However, the weak phase locking and frequency correlation observed in one animal (Rat 1) suggests that, under some circumstances, weak synchronization may occur.

## Discussion

We report on three main findings: First, in running rats the head moves rhythmically in the vertical plane with a frequency that depends monotonously on the running speed and lays in the theta band. Second, the amplitude of the theta rhythm depends on the phase lag between the head movements and the LFP oscillations. Third, the phase and frequency of the hippocampal theta rhythm are largely independent of the oscillatory dynamics of locomotion, at least when animals run for short periods of time.

The first finding could perhaps have been predicted from findings in other animals, especially humans, showing that the head oscillates vertically during locomotion in a speed-dependent manner [1], [2], [26], [30]. That the main frequency of these head oscillations are in the theta frequency band can be understood from three observations. First, the head moves up and down twice per step cycle (e.g. Fig. 2 in [30]). Second, the step cycle duration of rats running in the range of speeds observed in our study (50–100 cm/s) is about 200–300 milliseconds [21]. Third, head movements are regular when rats are trotting [25]. Our usage of miniature accelerometers (see also [31]) made it possible to uncover and precisely quantify these rhythmical movements and relate them to running speed and neurophysiological data recorded in freely behaving rodents. We found that both amplitude and frequency of the head oscillations increase almost linearly as a function of running speed. This implies that this oscillatory signal could be used to estimate instantaneous speed. In fact, we were able to reconstruct the distance traveled on a single trial from estimates of amplitude and frequency of the head oscillations (not shown). It is known that animals can use both inertial and other self-motion cues to navigate in their environment [3] and it seems likely that the prominent head oscillations we have characterized are also used for this purpose.

**Figure 7:**
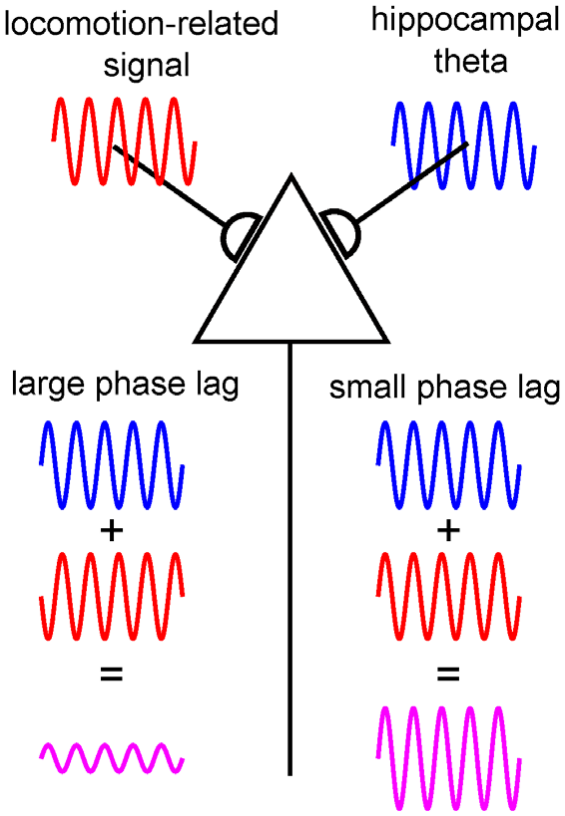
Interaction between locomotion-related inputs and hippocampal theta rhythm. Schematic illustration of the effect of phase on neuronal integration of two oscillatory signals. A neuron (triangle) receives two oscillatory inputs with similar frequency. One input (in red) is phase locked to the head acceleration signal. The other (in blue) is phase locked to the hippocampal theta rhythm. The amplitude of the outputs (violet traces) depends on the phase difference of the inputs.

The finding that the amplitude of the hippocampal theta rhythm depended on the phase lag between LFP and head oscillations was more unexpected. The effect was highly significant in the three animals and was not restricted to epochs during which the two signals have exactly the same frequency. It is also of sufficient magnitude to be observed in the raw data by comparing theta amplitude in trials in which head acceleration and LFP oscillations have different phase relation (e.g., Fig. 3) or in trials in which the phase differences between the two signals show a strong variability (e.g., Fig. 4A). Phase-dependent amplitude modulations could be generated through the simple mechanism illustrated in Fig. 7. In this scenario, neurons in the CA1 region are exposed both to the theta rhythm and a sensorimotor-related input locked to the head acceleration signal. These inputs have similar frequencies and are integrated in the neurons. The ‘output’ amplitude, e.g. of the membrane potential, therefore depends on the phase-relation between the two input signals. For the illustration in Fig. 7, the amplitude is enhanced when the two signals are in phase but the exact phase relation needed to increase (or decrease) the output amplitude would depend on the details of how the inputs are received (through which types of synapses, distance to the soma, etc.). It is clear, however, that if the inputs are in the same frequency range, their relative phase will generically influence the amplitude of the output. We discuss some possible pathways through which a ‘head-oscillation input’ could reach the hippocampus below. We note that, in our experiments, rats run faster than 50 cm/s and we can therefore only speculate on what happens at slower running speeds. We predict that, insofar as the locomotion is regular, the phase-dependent modulation of the hippocampal theta amplitude we report in this study should also be observed at slower running speeds.

The finding that the hippocampal theta oscillations are not entrained by the oscillatory dynamics of locomotion is reminiscent of recent results obtained by Berg et al. [32], showing that whisking, which also occurs within the 6–12 Hz frequency range, is not phase locked to the theta rhythm. Our result is supported by two complementary types of data analysis. First, we showed that in the three animals, despite the fact that the head acceleration and LFP oscillations share the same frequency band, the average coherence spectrum between the two signals does not have a significant peak in the theta band. Importantly, this result held true if the coherence analysis was restricted to periods during which the head movements displayed strong oscillations in the theta frequency band for at least half second. Second, we used a sliding windows method to detect the phase differences between the two signals. This method was applied during sustained oscillatory head movements and we only considered pairs of windows in which LFP and head oscillations had a similar frequency. In those restricted epochs, we found that the phase difference values were uniformly distributed in two out of three rats, while in one animal a weak but significant deviation from uniformity was found. Previous studies in rats have reported that bar pressing may be phase locked to the hippocampal rhythm [33], [34]. The lack of phase locking in our data support the more recent view that the frequency of theta oscillations is primarily set by intra-hippocampal dynamics [35], [36]. It should be noted however, that the weak phase locking we observed in one animal indicates that under some circumstances the theta rhythm can lock (weakly) to the locomotor rhythm. It could well be that if the rats would run for longer time intervals (in our experiments, typically, the rats crossed the maze in less than two seconds) more prominent phase and frequency locking would occur. In future experiment we will try to address this hypothesis by performing similar recordings in rats running on a treadmill.

Oscillatory sensorimotor input locked to the dynamic of head movements could modulate the hippocampal activity through a great number of sources. First, the vestibular system most likely generates a time-varying signal similar to the one recorded by the accelerometer we used [37], [38]. This signal could modulate hippocampal activity through a variety of polysynaptical pathways including the lateral mammillary nucleus, thalamus, subiculum and enthorinal cortex [39], [40]. Additionally, motor, proprioceptive and tactile (from the feet) inputs phase locked to the head oscillations can reach the hippocampus indirectly through the medial septum (reviewed in [5]). To test which of these “bottom-up” pathways mediates the amplitude modulation reported here is therefore a challenging task beyond the scope of the present study. Moreover, because all those sensorimotor activities are coordinated during locomotion, there is no simple way to selectively perturb one of them and investigate subsequent changes in synchrony between theta and head movements. For instance, pharmacological inactivation of the medial septum will lead to reduction of theta's amplitude that will preclude studying how it relates to head movements. It is nevertheless relevant to note that electrical stimulation of the vestibular system release acetylcholine in the hippocampus [41] and that rats with vestibular lesions have strongly decreased power of the hippocampal theta, an effect described several months after the lesion, when the animal runs almost normally [42].
